# *In vivo* knee biomechanics during badminton lunges at different distances and different foot positions by using the dual fluoroscopic imaging system

**DOI:** 10.3389/fbioe.2023.1320404

**Published:** 2023-12-21

**Authors:** Di Peng, Zheng Mao, Wang Zhang, Jinglun Yu, Shengnian Zhang

**Affiliations:** Key Laboratory of Exercise and Health Sciences of Ministry of Education, School of Exercise and Health, Shanghai University of Sport, Shanghai, China

**Keywords:** fluoroscopy, knee kinematics, badminton, lunges, sports injury

## Abstract

**Background:** Lunges are common in badminton. Distance and foot position affect knee joint loadings under lunges, which are closely related to knee injury incidence. Investigations involving dynamic knee motion *in vivo*, kinetics, and muscle activation in lunges, especially during lunges of different distances and foot positions, are instrumental for understanding knee performance and injury risks of players.

**Methods:** A total of 10 experienced badminton athletes (10 females; height, 164.5 ± 5.0 cm; weight, 59.3 ± 6.0 kg; and age, 22 ± 1.0 years) were recruited. By using a high-speed dual fluoroscopic imaging system, Qualisys motion capture system, Kistler force plate, and Delsys electromyography simultaneously, data were collected during players’ 1.5 times leg length lunge, the maximum lunge, and the maximum lunge while the foot rotated externally. Magnetic resonance and dual fluoroscopic imaging techniques were used to analyze the *in vivo* knee kinematics.

**Results:** Compared with the 1.5 times leg length lunge, knee flexion for the maximum lunge increased significantly (*p* < 0.05). The anterior–posterior ground reaction force (GRF) and vertical GRF of the maximum lunge were significantly higher than those of the 1.5 times leg length lunge. During the two different foot position lunges with the maximum distance, the posterior translation of knee joint was larger (*p* < 0.05) when the foot rotated externally than the normal maximum lunge. Moreover, the anterior–posterior GRF and vertical GRF increased significantly when the foot rotated externally. Significant differences were observed in valgus–varus rotation torque and internal–external rotation torque of the knee joint under the two distance lunges and two foot position lunges (*p* < 0.05). No significant difference was found in knee muscle activation during the two distance lunges and during the two foot position lunges.

**Conclusion:** High knee torque and compressive loadings with increasing lunge distance may cause knee injuries in badminton. When lunging in the external foot rotation under the maximum distance, high quadriceps force and posterior tibia translation force could result in knee injuries among badminton players.

## 1 Introduction

Badminton is a highly demanding game characterized by high-intensity, intermittent actions (Phomsoupha and Laffaye, 2015). The lunge is one of the most frequently used movements in badminton (Shariff et al., 2009). The sudden and repeated lunge is relevant to badminton injuries, and it is especially common in overuse injuries. The prevalence of overuse injuries in badminton matches is about 36%, and the majority of injuries occur in the lower extremities (Jacobson et al., 2005). The knee joint is a highly documented injured site in the lower extremities of badminton players (Jørgensen and Winge, 1990).

The incidence of knee injuries is closely associated with strenuous impact force (Dos'Santos et al., 2018). Large vertical and horizontal loadings at the heel contact phase under lunges generate a high joint torque on the knee joint, contributing to anterior cruciate ligament (ACL) injuries (Abián-Vicén et al., 2012; Lam et al., 2017). In addition, repeated and accumulated loadings can cause overuse knee injuries (Shariff et al., 2009; Boesen et al., 2011), such as patellar tendinosis or patellofemoral joint pain syndrome (Jørgensen and Winge, 1990; Kimura et al., 2010; Lam et al., 2017).

High loadings influence the dynamic knee motion during lunges, resulting in large anterior tibia translation (ATT) and large varus rotation (Hewett et al., 2005). Notably, large tibia internal rotation can significantly strain the ACL (Markolf et al., 1995) and increase the potential risk of the knees. Abnormal movements of the knee joint can also lead to meniscus tear and cartilage degeneration (Riemann and Lephart, 2002). Abnormal kinematics and consequent abnormal cartilage deformation within the joints initiate knee osteoarthritis (Felson et al., 2000; Sharma et al., 2001).

The left-forward lunge is considered a critical maneuver for badminton biomechanics because of its significantly higher external and insole loadings than other lunge directions (Dos'Santos et al., 2018; Hong et al., 2014). Besides lunges of different directions, players also perform lunges of various distances to move into the best position for varying offensive and defensive shots in a match. The lunge distance is the distance from which the player begins to lunge until the last footstep before hitting the shuttlecock, which indirectly reflects the players’ lunging performance. Lunge distances have been linked with the leg length of badminton players, such as 1.5 times leg length (Kuntze et al., 2010) and 2.5 times leg length (Hong et al., 2014; Huang et al., 2014). The maximum lunging distance is also analyzed to assess the players’ maximal lunge capabilities (Hu et al., 2015). Thus, increased attention to impact loading characteristics during the left-forward lunge of different distances is warranted. Players show different foot positions frequently during lunges in competition, especially when the lunge distance increases to the maximum. No clear indication is given concerning the optimal angle of foot placement in badminton lunges. However, foot placement and body alignment play a significant role in balance and mobility (Paillard et al., 2006). Furthermore, foot position affects the muscle activation level of the lower limbs, especially affecting the activation of quadriceps and hamstrings of knee joints (Wang et al., 1990). Thus, lunge distance and foot position are suggested to be essential for lunge performances and badminton injuries.

Accurate quantification of six degrees of freedom (DOF) knee kinematics during lunges is critical to promote sports performances and recognize abnormal motion relevant to injury mechanisms of badminton players. In general, the external joint moments and 3D motion of players were previously used as surrogate variables to analyze lunge characteristics (Huang et al., 2014; Hu et al., 2015). How precisely the femur and the tibia translate or rotate under left-forward lunges in badminton has been scarcely reported *in vivo*, as well as under left-forward lunges of different distances and different foot positions. The dual fluoroscopy imaging system (DFIS) enables the accurate monitoring of *in vivo* bone motion without soft tissue artifacts (Li et al., 2013). DFIS is a new non-invasive bone movement tracking system that has been used for 3D kinematic measurement. It has been validated with submillimeter and sub-degree accuracy in translation and rotation (Yack et al., 1994).

The current study used DFIS to measure *in vivo* knee translations and rotations of badminton players during left-forward lunges of two distances and two foot positions, as well as to investigate the kinetics and knee muscle activation of players performing lunges. The primary objective of this study was to investigate the *in vivo* knee performance in left-forward lunges and examine the knee biomechanics and potential knee injury risks in badminton lunges. The second objective was to explore the effects of distance and foot position on the knee performance of badminton players in left-forward lunges. We hypothesized that femoral valgus rotation, posterior translation, and distal translation may increase in the maximum lunge. We observed distinct knee performances of kinetics and muscle activation in players doing left-forward lunges under two distances and two foot positions.

## 2 Materials and methods

### 2.1 Study protocol

Ten experienced badminton athletes were recruited in this study (10 females; height, 164.5 ± 5.0 cm; weight 59.3 ± 6.0 kg; and age, 22 ± 1.0 years) according to experience. The players were all active participants in singles badminton competitions at the university level and had at least 5 years of badminton experience. All participants had no history of injuries or surgeries in their lower limbs. The right leg must be their dominant leg, and we verified this information by observing which leg the subjects used when they were asked to kick a football. All players were free from any lower extremity injuries for at least 6 months prior to the experiment. The task encompassed three components: the left-forward lunge at a distance of 1.5 times the individual’s leg length, the individual’s maximum left-forward distance, and the maximum left-forward lunge with foot external rotation. This study was approved by the local ethics committee, and all participants signed an informed consent form.

**FIGURE 1:**
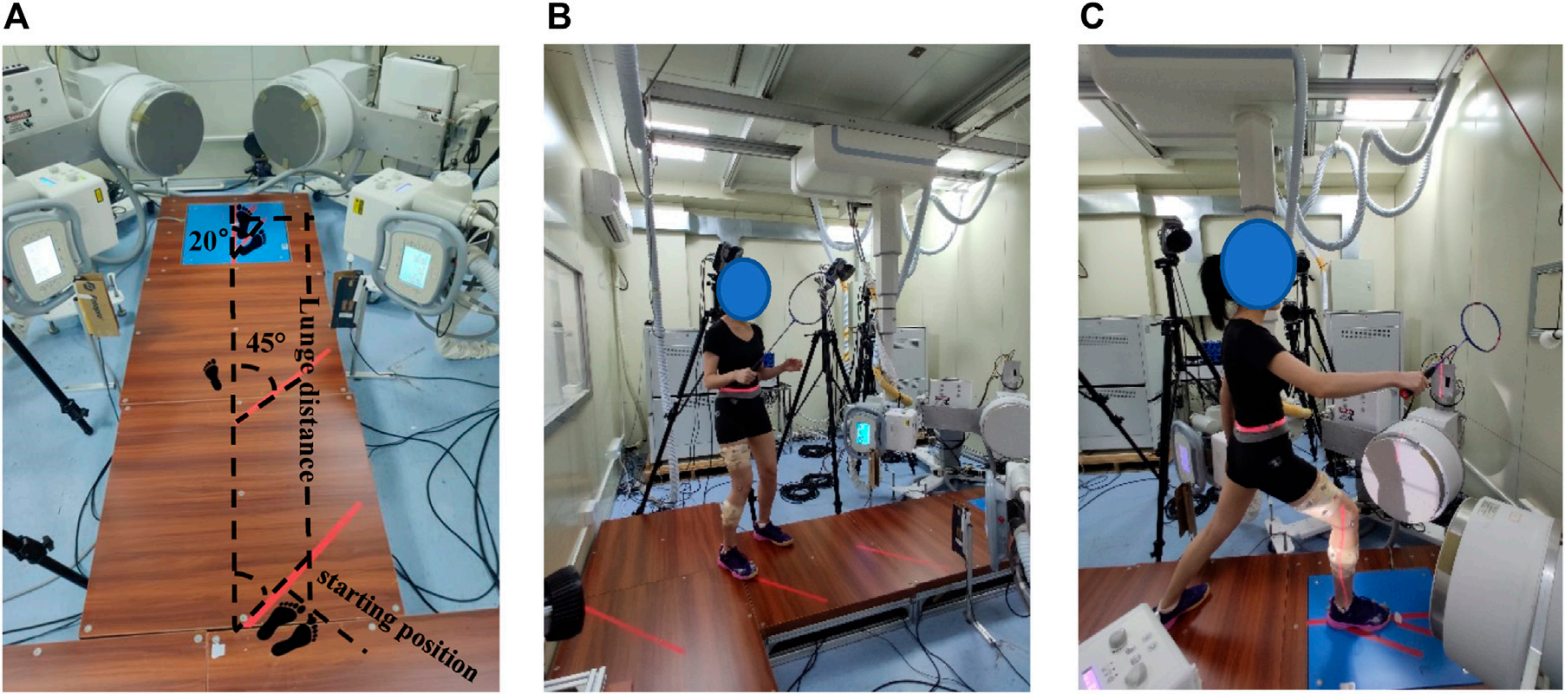
**(A)** Experimental set-up. **(B)** Subject prepared a standardized initial position at the starting position. **(C)** Subject performed the 1.5 times *leg length left-forward lunge.

High-speed dual-plane fluoroscopic imaging system, force plate (60 cm × 50 cm × 10 cm, Kistler Corporation, Winterthur, Switzerland) with 1,000 Hz, motion analysis system of eight cameras (Oqus700+, Qualisys, Switzerland) with 200 Hz, and Delsys wireless surface electromyography with 2000 Hz were used simultaneously in the study (Figure 1). DFIS images were acquired using two commercially available BV Pulsera C-arms and 40 cm image intensifiers with a sampling frequency of 100 Hz, 60–70 kVp, 60–70 mA, and 1 ms pulsed exposure during lunging.

### 2.2 Data collection

The subjects’ right knee was scanned by a 3-T magnetic resonance imaging (MRI) scanner before the trial (voxel size: 0.3 mm × 0.3 mm × 1.0 mm, FOV read: 180 mm, and slice thickness: 1 mm). Bone contours from MRI images were extracted to reconstruct the 3D geometry of the femur and tibia in Avizon 2019.1 (Thermo Fisher Scientific) software. These high-resolution images were manually segmented to create a finite element mesh of the bones of the femur and tibia.

The basic data of the participants (i.e., age, gender, body weight, and height) were collected. The leg length (vertical distance from the anterior superior iliac spine to the ground) was also measured. One experimenter explained the procedure to subjects, instructed them to perform warm-up exercises for 10 min, including stretching and jogging, and familiarized them with left-forward lunges.

Eight surface electrodes were set to the right lower limb’s rectus femoris, vastus lateral, vastus medial, biceps femoris, semitendinosus, lateral gastrocnemius, medial gastrocnemius, and tibialis anterior. Subsequently, we tested the maximum voluntary contraction (MVC) of the muscles when subjects were asked to resist to complete isometric contraction for 5–8 s, to normalize the EMG data of each muscle. Reflective markers were placed over the participants’ right legs according to a previous segment model (Lam WK et al., 2017).

All participants were asked to wear Decathlon BS 530 badminton shoes during lunge trials, and this shoe is often used in badminton training and competitions. Lunge tasks included a lunge distance equal to 1.5 times the length of an individual’s leg, individual’s maximum distance, and maximum lunge with foot external rotation. The interval between groups was 30 s. Compared with the maximum lunge, the femur rotated externally, the right toe was far from the body, and the foot deviation angle increased by 20° in the maximum lunge with foot external rotation (Figure 1). Both tasks were required to lunge with the maximum distance. That 20° deviation angle was based on the angle of players’ foot external rotation in training and matches, as well as the suggestions from coaches.

Before the actual trial, each participant familiarized themselves with the left-forward lunge. Nine successful lunges (one block of three trials) should be collected successfully. The participants prepared a standardized initial position at the starting point holding the badminton racket, extended their dominant leg as far as possible, and landed on the force platform while hitting the shuttlecock. After hitting, participants were required to return to the starting position quickly. A successful lunge trial consisted of maximum effort, correct foot placement at the starting and ending line, contact of the dominant leg with the center of the force plate, the hit of the shuttlecock, and rapid recovery. Participants performed each lunge in one step. Lunge start and finish points were marked out as visual references. The start point was 45° with respect to the longitudinal axis of the force platform (Figure 1).

### 2.3 Data reduction

#### 2.3.1 3D kinematic and kinetic data

Raw kinematic and kinetic data were exported to Visual3D software (C-Motion Inc., Rockville, MD, United States) and filtered using low-pass Butterworth filters with cutoff frequencies of 10 and 50 Hz, respectively. The ground reaction forces were normalized by body weight (BW). The contact phase of the lunge was identified as the period from initial heel contact of the landing foot to toe off, starting with vertical ground reaction force (VGRF) excessed to 10N until the right toe off the ground as determined by the force plate.

#### 2.3.2 6DOF characteristics of the tibiofemoral joint

**FIGURE 2:**
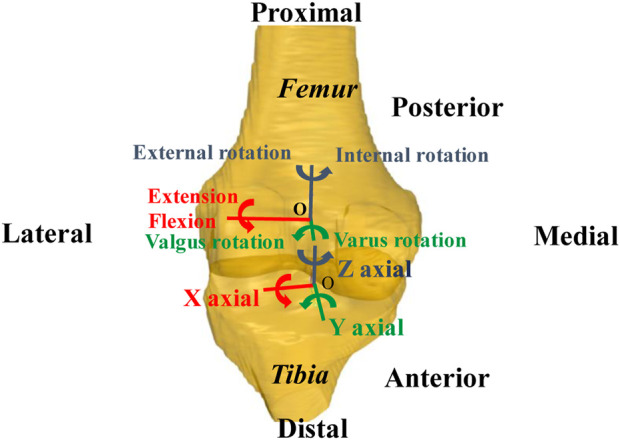
Coordinate systems of femur and tibia.

**FIGURE 3:**
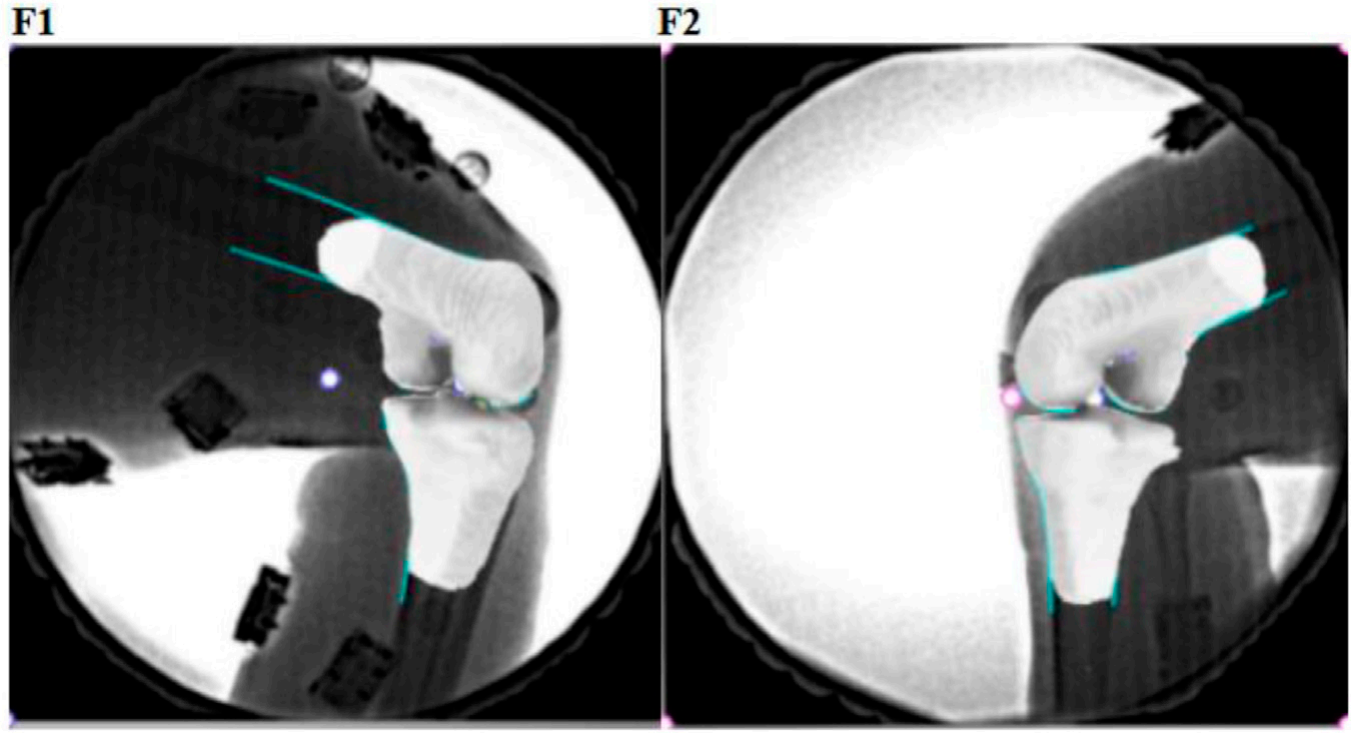
Dual-plane fluoroscopic images (F1 and F2) with 3D knee models were combined to reproduce the knee position.

Coordinate systems were established to investigate 6DOF kinematics of the tibiofemoral joint (Most et al., 2004; Kai et al., 2014). The *X*-axis of the femoral coordinate system was constructed by connecting circle centers of medial and lateral condyles with a line (Figure 2). The *Z*-axis was drawn parallel to the posterior wall of the femoral shaft in the sagittal plane. The *Y*-axis was perpendicular to the *X*-axis and long axes. The middle point of the *X*-axis was defined as the origin of the femoral coordinate system. For that tibia coordinate system, the *X*-axis was drawn parallel to the posterior edge of the tibial plateau. The *Z*-axis was the long axis of the tibial shaft through the middle of tibial spines. The *Y*-axis was perpendicular to the *X*-axis and *Z*-axis. The midpoint of the connecting line of the tibial plateaus was defined as the origin of the tibial coordinate system. Using Rhino 6.0 SR5 software (Robert McNeel and Associates, United States), the virtual DFIS setup environment was established and bony landmarks were sketched. The 3D bone model was optimally positioned to match the projection contour of the DFIS image, and we calculated the 6DOF kinematics of the tibiofemoral joint with the tibial and femoral coordinate systems established (Figure 3). The *in vivo* knee motion was described as the relative motion of the tibial coordinate system with respect to the femoral coordinate system.

#### 2.3.3 Electromyography data

The electromyography (EMG) data were processed in Delsys EMGworks Analysis 4.2.0.0 software (Calculation Toolkit 1.5.2.0). Raw EMG signals were filtered and smoothed using a Butterworth filter at the band-pass frequency (10–400 Hz) to attenuate artefacts. After adjusting baseline and full-wave rectification, the root mean square (RMS) and integrated electromyography (IEMG) amplitude of each signal were calculated. The RMS and IEMG data were normalized to the MVC of each muscle. The muscle pre-activation and post-activation were defined as the muscle activity level of 50 ms before and after touchdown, and the co-activation was the ratio of agonist muscles against antagonistic muscles.

### 2.4 Statistical analysis

The normality of the variables was tested with a Shapiro–Wilk test, and we found that all variables were normally distributed. Repeated-measures ANOVA (*α* = 0.05) and Turkey’s post-test were used to compare differences in 6DOF knee kinematics among the three left-forward lunges. The paired-T test was used to compare the differences *in vivo* knee 6DOF kinematics (antero–posterior tibial translation, mediolateral tibial translation, internal–external tibial rotation, and varus–valgus tibial rotation), kinetics, and muscle activities between the two distance lunges and two foot position lunges. Parameters are shown by mean ± standard deviation. Significance analysis was performed by IBM SPSS statistics 26.0 (IBM Inc., Chicago, IL, United States) software, and the significance *α* level was set at *p* < 0.05.

## 3 Results

### 3.1 6DOF kinematics of tibiofemoral joint

For the two distance lunges, compared with the 1.5 times leg length lunge, the flexion and varus rotation of the tibiofemoral joint increased significantly by 4.5° (*p* = 0.014) and 1.6° (*p* = 0.033) during the maximum left-forward lunge. In particular, the varus rotation significantly increased by 1.4° (*p* = 0.048) in the braking phase of the maximum lunge compared with that of the 1.5 times leg length lunge. The flexion and varus rotation increased by 4.5° (*p* = 0.033) and 1.9° (*p* = 0.048), respectively, in the recovery phase under the maximum distance (Table 1).

**TABLE 1:** Six degrees of freedom of the tibiofemoral joint in the phases of three lunges.

6 degrees of freedom	1.5 times leg length lunge		The maximum lunge		The maximum lunge with foot external rotation	
	Braking	Recovery	Braking	Recovery	Braking	Recovery
Flexion (+)Extension (−) (deg)	57.3 ± 7.8	46.6 ± 6.3	61.8 ± 9.6	51.1 ± 7.8*	64.3 ± 8.9	52.2 ± 7.0
Valgus (+)Varus (−) rotation (deg)	−7.2 ± 1.9	−8.5 ± 2.9	−8.6 ± 2.8*	−10.3 ± 3.4*	−7.8 ± 2.8	−8.2 ± 4.0
External (+)Internal (−) rotation (deg)	3.2 ± 2.0	3.1 ± 1.8	2.8 ± 1.6	2.6 ± 1.5	3.9 ± 1.2	3.9 ± 2.7
Lateral (+)Medial (−) translation (mm)	−2.1 ± 0.6	1.30 ± 1.2	−1.9 ± 1.2	1.2 ± 0.9	−1.4 ± 0.4	0.7 ± 0.3
Anterior (+)Posterior (−) translation (mm)	−7.0 ± 3.6	−7.6 ± 4.0	−7.1 ± 3.4	−7.9 ± 4.1	−6.0 ± 2.9	−7.0 ± 3.9
Proximal (+)Distal (−) translation (mm)	26.4 ± 2.4	26.8 ± 2.8	26.1 ± 3.0	26.4 ± 3.0	26.0 ± 3.2	26.2 ± 3.1

Denotes the variable that was significantly different under lunges at two distance and two foot position, significant *p* values (*p* < 0.05); SD, standard deviation. The braking phase was from the initial contact to the maximum knee flexion, and the recovery phase was from the maximum knee flexion time to the right toe off the ground.

**FIGURE 4:**
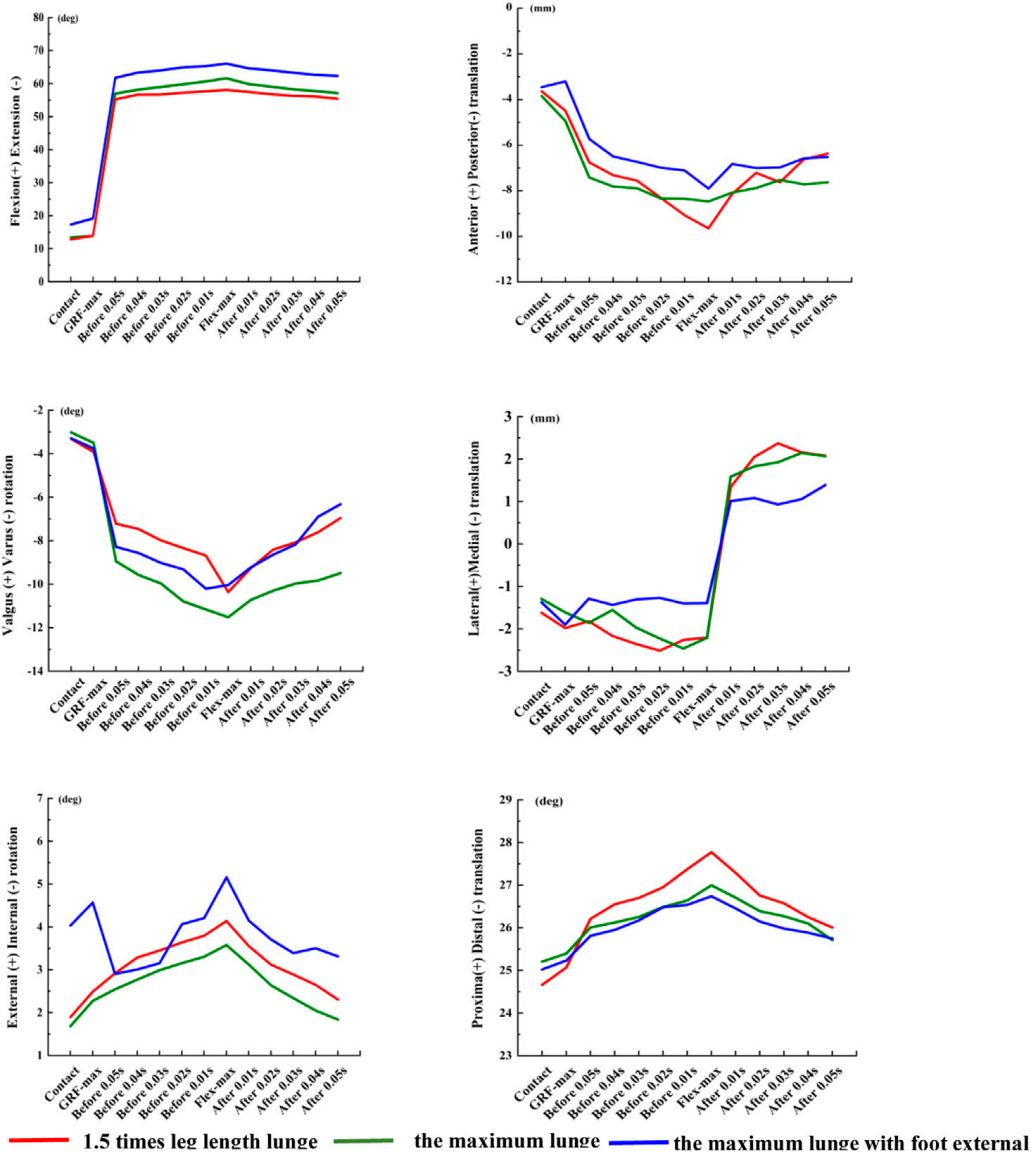
Six degrees of freedom of the knee joint in the characteristic points of three left-forward lunges. Contact, the initial moment of foot contact force plate; GRF-max, the point of peak vertical reaction force; Flex-max, the moment of the knee maximum flexion; Before/After 0.05 s, Before/After the maximum knee flexion 0.05 s; Before/After 0.04 s, Before/After the maximum knee flexion 0.04 s; Before/After 0.03 s, Before/After the maximum knee flexion 0.03 s; Before/After 0.02 s, Before/After the maximum knee flexion 0.02 s; Before/After 0.01 s, and Before/After the maximum knee flexion 0.01 s.

In terms of the characteristic points during the two distance lunges, compared with the 1.5 times leg length lunge, the flexion increased significantly by 4.0° (*p* = 0.014) at the initial contact moment in the maximum lunge (Figure 4), and the flexion increased significantly at the point of initial GRF peak by 5.2° (*p* = 0.01). The varus rotation of the knee joint increased significantly both from 0.03 s before the maximum knee flexion to 0.01 s before the maximum knee flexion (*p* < 0.05). The varus rotation increased significantly from 0.03 s after the maximum knee flexion to 0.05 s after the maximum knee flexion (*p* < 0.05). The posterior translation of the femur relative to tibia significantly increased at 0.04 and 0.05 s after maximum knee flexion (*p* < 0.05).

During the two foot position lunges, the posterior translation of the femur relative to the tibia decreased significantly (*p* < 0.05) in the maximum lunge with foot external rotation, especially at 0.02 s, 0.01 s before the maximum knee flexion and 0.01 s after the maximum knee flexion (Figure 4). When the foot position changed, the lateral translation of the femur relative to the tibia decreased significantly to 1.09 mm (*p* = 0.034) at 0.04 s after the maximum knee flexion.

### 3.2 Kinetics

**FIGURE 5:**
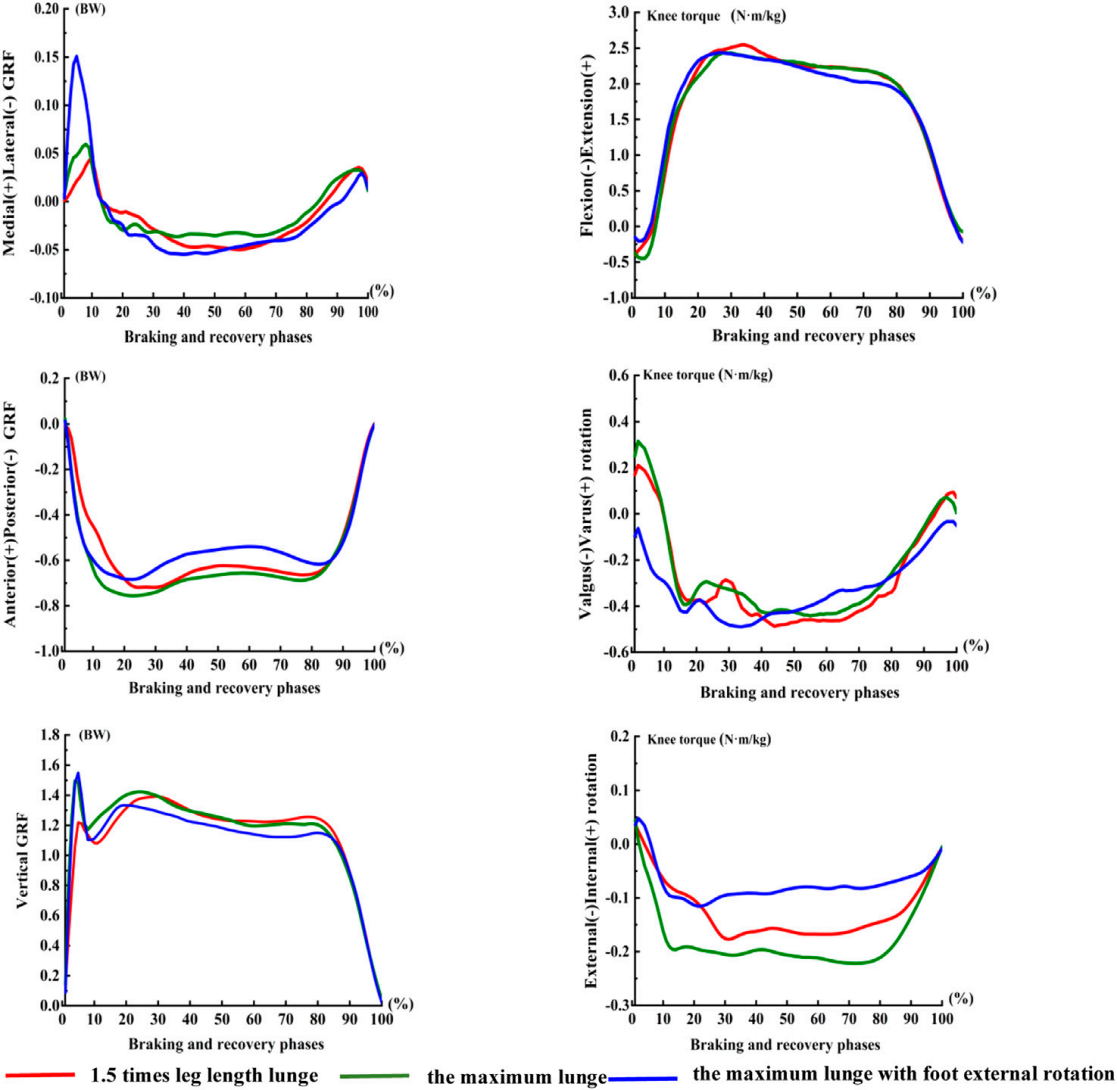
Torque of knee in the phases of three left-forward lunges. The braking and recovery phase of the lunge was defined as the period from initial heel contact of the landing foot to toe off, starting with VGRF excessed to 10N until the right toe off the ground determined by the force plate.

During the two distance lunges, for the maximum lunge, the VGRF significantly increased 0.02BW (*p* = 0.013; Figure 5), the anterior-posterior GRF significantly increased 0.05BW (*p* = 0.001), and the medial-lateral GRF significantly decreased 0.01BW (*p* = 0.001). During the two foot position lunges, when the landing foot rotated externally, the vertical GRF decreased significantly (*p* = 0.023), the anterior–posterior GRF significantly decreased by 0.07BW (*p* = 0.022), and the medial–lateral GRF increased by 0.01BW significantly (*p* = 0.002).

We noted significant differences in valgus–varus rotation torque and internal–external rotation torque of the knee joint under the two distance lunges and two foot position lunges (*p* < 0.05). The lunge distance and foot position had no significant effect on knee power in the left-forward lunge.

### 3.3 Muscle activation

**FIGURE 6:**
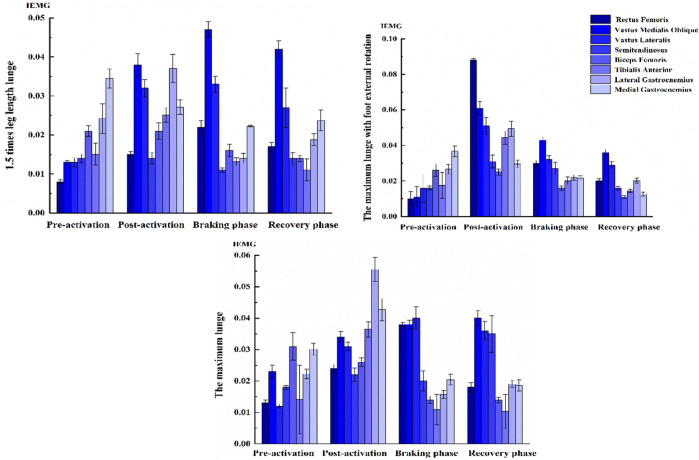
Muscle activation level of knee in left-forward lunges. The time of muscle pre-activation was defined as 50 ms before touchdown, the time of muscle post-activation was defined as 50 ms after the maximum knee flexion, the braking phase was from the initial contact to the maximum knee flexion, and the recovery phase was from the maximum knee flexion time to the right toe off the ground.

Under the two distance lunges, the muscle activities of the knees in pre-activation and post-activation were higher in the maximum lunge than in the 1.5 times leg length lunge. Under the two foot position lunges, the activity level of thigh and calf muscles in the pre-activation and post-activation of the maximum distance with foot rotated externally were higher than those of the maximum lunge. Under the two distance and two foot position left-forward lunges, no significant difference was observed in knee muscle co-activation, pre-activation, and post-activation (Figure 6).

## 4 Discussion

Compared with the 1.5 times leg length lunge, the flexion and varus rotation of the knee joint significantly increased under the maximum lunge. The posterior tibia translation and the knee muscle co-activation increased under the maximum distance with the foot external rotation than the maximum lunge. This result supported our hypothesis that the increased lunge distance and the increased foot external rotation would increase the flexion and varus rotation of the knee joint.

Previous studies showed that the flexion of the knee increases and the tibia moves anteriorly during static lunges (Komistek et al., 2003). The internal tibial rotation increases sharply during low knee flexion tasks (Qi et al., 2013). When the knee flexion was at 30°–120°, the tibia rotated internally constantly (Moro-oka et al., 2008), the posterior translation of the femur related to the tibia moved to 13.3 ± 3.2 mm, and the tibia rotated in varus by 4.1° ± 3.6° (Qi et al., 2013). However, the anterior–posterior translation of the femur was larger than the results of this study, and the varus–valgus rotation was small, which was relevant to the static one-leg squat trial *versus* the dynamic left-forward lunges.

The kinematics of the knee during functional tasks could be influenced by external forces, joint position, and the balance of active and passive contributory forces across the knee (Myers et al., 2012). The 6DOF knee motion varied during functional activities to fulfill the tasks or to optimize the motor output efficiency. Studies confirmed that ATT significantly increases as demand on the quadriceps increases, and landing produces significantly greater peak ATT than walking and unweighted full extension (Myers et al., 2012). ATT increases with knee flexion during a lunge activity (Defrate et al., 2006). With the lunge distance increasing in this experiment, the flexion and varus rotation of the knee joint significantly increased during the maximum lunge than those during the 1.5 times leg length lunge. Thus, the knee regulated stability mechanically by the inherent geometry of bones and soft tissue stiffness among tasks.

Persistent abnormal knee kinematics could be a putative factor in the degeneration of cartilage (Gill et al., 2009). The increased varus shear force and the anterior tibia drawer force during the maximum lunge may lead to the excessive tension of ACL of badminton players. We knew that ACL plays an important role in maintaining knee joint stability as the main passive restraint of the knees during activities (Ahmed et al., 1992). However, women have lower torsional knee joint stiffness than men in response to combined rotational loads (Hsu et al., 2006). With the increasing demands on the thigh musculature in activities, women cannot generate a sufficient reaction moment against the externally applied internal rotation moment of the shank relative to the femur (Kiriyama et al., 2009). These findings illustrated that the potential risk of knee injury among female players is high when lunging in foot external rotation with multiple loadings.

When the foot rotated externally under the maximum distance, the posterior movement of the femur relative to the tibia significantly decreased at 0.02 s before, 0.01 s before, and 0.01 s after the maximum knee flexion than the maximum lunge. Posterior cruciate ligament (PCL) played a key role in limiting tibia posterior translation. The strong external load exerted on the proximal tibia under knee flexion resulted in excessive posterior movement of the tibia, which may eventually lead to PCL injuries. When the tibia moved posteriorly at knee 90° flexion, the internal torque and varus rotation moment simultaneously acted on the knee joint, which may result in non-contact PCL rupture (Markolf et al., 1996). In addition, with the increased foot external rotation during the maximum lunge, the lateral translation of the femur relative to the tibia decreased significantly at 0.04 s after the maximum knee flexion. The rotation of lower limbs could change the force line of the ankle joint, making it move medially during external rotation (Riemann et al., 2011).

The first valley and the second peak of the sagittal plane knee moment in the maximum lunge were larger than those of 1.5 times leg length lunge, which may be due to the increased knee work with large lunge distance. The knee did negative work in the sagittal plane during the braking phase and then began to do positive work after the maximum knee flexion. The muscle completed eccentric contraction when braking and performed negative work to absorb human energy. In the recovery phase, the muscle performed concentric contraction to complete the lower limb push and extend for the next hit. In addition, VGRF at the maximum distance increased significantly than that at the 1.5 times leg length lunge, especially at the first peak of VGRF. Strenuous impact forces in badminton lunges are closely related to knee injury incidence, especially ACL injuries (Abián-Vicén et al., 2012; Dos'Santos et al., 2018; Lam et al., 2017).

In terms of muscle activation, the pre-activation levels of the quadriceps and hamstrings increased as the lunge distance increased. This result may be related to the large VGRF under the maximum lunge distance. High muscle pre-activation modulated the 6DOF knee kinematics mechanically to maintain stability. Thus, the thigh muscles could regulate the translation and rotation of the tibia within a limited range under different functional activities. The preparatory and reflexive muscle activations of the quadriceps and hamstrings were integral during activities, such as walking and landing, in providing knee joint stability to control translations and rotations (Riemann and Lephart, 2002). This phenomenon may also help explain why the ATT and internal tibia rotation increased in previous studies where efforts had been made to minimize thigh musculature pre-activation and hamstring co-activation during trials (Shariff et al., 2009).

However, co-activation in the thigh and calf increased during the maximum distance. The knee muscles maintained strong activities during the maximum distance to strengthen joint stability. Studies have reported that quadriceps contraction applies an anterior shear force on the proximal end of the tibia through the patellar tendon (Wall et al., 2012). As the quadriceps force increases, so does the anterior shear force, ATT, and ACL force (DeMorat et al., 2004). A modeling study of ACL function suggested that quadriceps force and compressive force acting at the tibiofemoral joint contribute greatly to the total load on the ACL (Pflum et al., 2004).

When the knee is in an extended position, and in the absence of hamstring co-activation, the quadriceps reportedly produce sufficient ATT to tear the ACL (DeMorat et al., 2004). Combined with quadriceps muscle force or anterior shear force, the increased ACL load is higher under the knee internal rotation torque than under the external rotation torque (Markolf et al., 2004). In competitive sports, eccentric quadriceps contraction during landing and the resulting shear force on the proximal end of the tibia have been reported to be risk factors for ACL injury (Hewett et al., 2006). Compared with the maximum distance, the pre-activation of the thigh and calf muscles increased under foot external rotation. The abnormal muscle force of the knee could contribute greatly to the total load on the ACL. The foot position affected the activity level of the quadriceps, and the hamstrings were also activated differently when changing foot position (Wang et al., 1990). Moreover, the activation of the medial hamstring muscle was observed to increase significantly during landing in tibia internal rotation than in external rotation (Mohamed et al., 2003). The lateral translation of the tibiofemoral joint decreased significantly under the maximum lunge with foot external rotation, which may induce abnormal medial translation of the tibia. The internal structures of the knee that constrained this translation were biceps femoris muscle contraction or ligaments in tension. Thus, in this study, we observed an increase in activation of biceps femoris when lunging in foot external rotation.

In this study, a small sample size was used. Two-step or three-step lunges were excluded from this study because of the limited space of the laboratory. Given the limitation of the shooting perspective of DFIS, the initial contact and the maximum flexion of the knee cannot be captured at the same time, so these data were collected separately to analyze the full motion phase. This study focused on *in vivo* knee bone movement by DFIS, so further investigations on the mechanical characteristics of the internal tissues of the knee joint are necessary through modeling and finite element analysis (Sun et al., 2022; Cen et al., 2023; Song et al., 2023).

## 5 Conclusion

This study described the 6DOF knee motion characteristics. It also revealed the kinematics and knee muscle activation during badminton lunges at different distances and different foot positions. This study revealed two key findings: 1) the higher vertical GRF, the larger varus rotation and flexion of the knee joint during the maximum lunge; thus, high knee torque and load may cause a potential risk injury with the increased distance; 2) during the two foot position lunges, the increased posterior tibia translation may increase the risk of knee injury with the foot external rotation. These data may provide theoretical guidance for lunge biomechanics to promote sports performance and prevent injury among badminton athletes.

## Data Availability

The original contributions presented in the study are included in the article/Supplementary Material, further inquiries can be directed to the corresponding author.
