# Supplementary material for: In vivo knee biomechanics during badminton lunges at different distances and different foot positions by using the dual fluoroscopic imaging system
Source: Front Bioeng Biotechnol. 2023 Dec 21;11:1320404. doi: 10.3389/fbioe.2023.1320404 (PMC10768190; doi:10.3389/fbioe.2023.1320404)
Supplement: Supplementary file 2 [file Table1.DOCX]

**Table 1.** Six degrees of freedom of the tibiofemoral joint in the phases of three lunges

| 6 degrees of freedom | 1.5 times leg length lunge | | the maximum lunge | | the maximum lunge with foot external rotation | |
| --- | --- | --- | --- | --- | --- | --- |
|  | Braking | Recovery | Braking | Recovery | Braking | Recovery |
| Flexion(+)Extension(-)  (deg) | 57.3 ± 7. 8 | 46.6 ± 6.3 | 61.8±9.6 | 51.1±7.8* | 64.3 ± 8.9 | 52.2 ± 7.0 |
| Valgus(+)Varus(-) rotation(deg) | -7.2 ± 1.9 | -8. 5 ± 2.9 | -8.6 ± 2.8* | -10.3 ± 3.4* | -7.8 ± 2.8 | -8.2 ± 4.0 |
| External(+)Internal(-)  rotation(deg) | 3.2 ± 2.0 | 3.1 ± 1.8 | 2.8 ± 1.6 | 2.6 ± 1.5 | 3. 9 ±1.2 | 3.9 ± 2.7 |
| Lateral(+)Medial(-) translation(mm) | -2.1 ± 0.6 | 1.30 ± 1.2 | -1.9 ± 1.2 | 1.2 ± 0.9 | -1.4 ± 0.4 | 0.7 ± 0.3 |
| Anterior(+)Posterior(-)  translation(mm) | -7.0 ± 3.6 | -7.6 ± 4.0 | -7.1 ± 3.4 | -7.9 ± 4.1 | -6.0 ± 2.9 | -7.0 ± 3.9 |
| Proximal(+)Distal(-) translation(mm) | 26.4 ± 2.4 | 26.8 ± 2.8 | 26.1 ± 3.0 | 26.4 ± 3.0 | 26.0 ± 3.2 | 26.2 ± 3.1 |

* denotes the variable that was significantly different under lunges at two distance and two foot position, significant *P* values (*P* < 0.05); SD, standard deviation. The braking phase was from the initial contact to the maximum knee flexion, and the recovery phase was from the maximum knee flexion time to the right toe off the ground.
