# Supplementary material for: In vivo knee biomechanics during badminton lunges at different distances and different foot positions by using the dual fluoroscopic imaging system
Source: Front Bioeng Biotechnol. 2023 Dec 21;11:1320404. doi: 10.3389/fbioe.2023.1320404 (PMC10768190; doi:10.3389/fbioe.2023.1320404)
Supplement: Supplementary file 4 [file DataSheet1.DOCX]

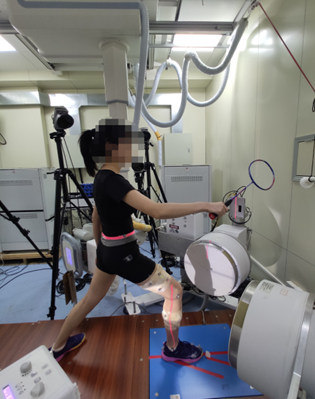

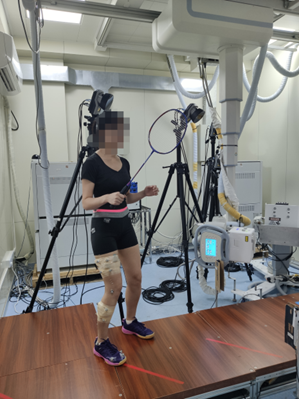

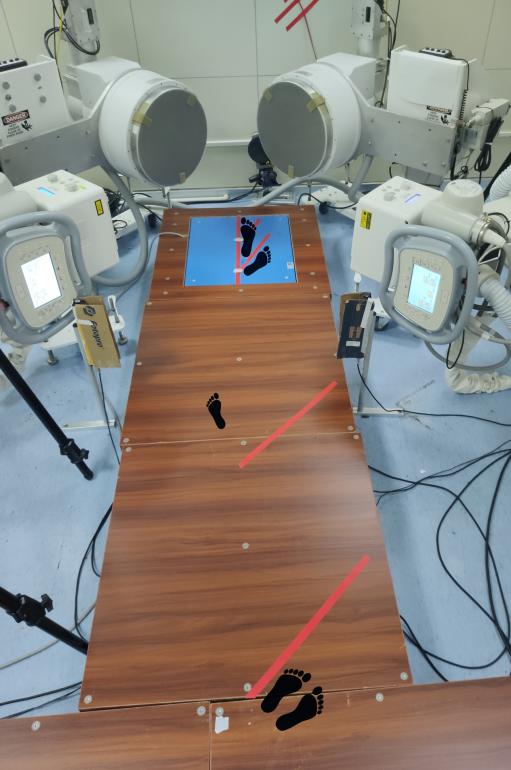


**a**

**c**

**b**

**20°**

**Lunge distance**

**45°**

**Figure 1.** (a) Experimental set-up. (b) Subject prepared a standardized initial position at the starting position. (c) Subject performed the 1.5 times *leg length left-forward lunge.

**starting position**


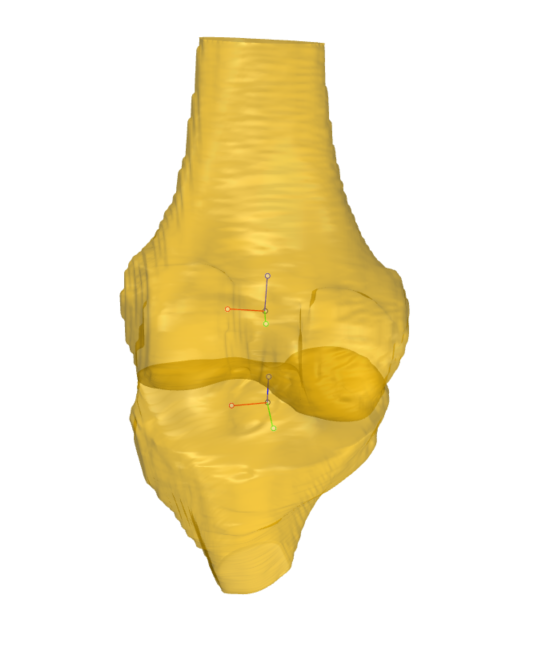


**Medial**

**Proximal**

**Distal**

**Lateral**

**Y axial**

**Z axial**

O

**O**

**Anterior**

**Posterior**

**Varus rotation**

**Valgus rotation**

**Extension**

**Internal rotation**

**External rotation**

**X axial**

**Figure 2.** Coordinate systems of femur and tibial.

**Flexion**

**
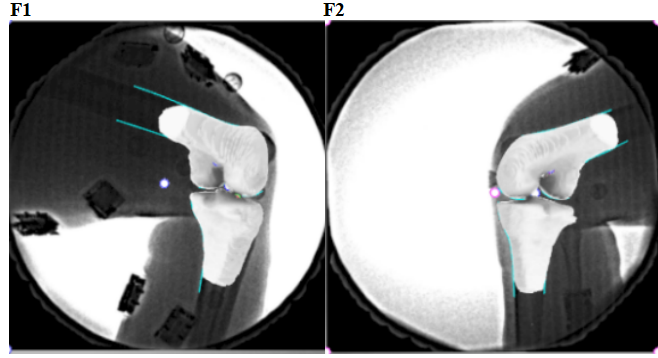
**

**Figure 3.** Dual-plane fluoroscopic images (F1 and F2) with 3D knee models were combined to reproduce the knee position.

















**1.5 times leg length lunge**

**the maximum lunge**

**the maximum lunge with foot external roation**

**Figure 4.** Six degrees of freedom of the knee joint in the characteristic points of three left-forward lunges. Contact, the initial moment of foot contact force plate; GRF-max, the point of peak vertical reaction force; Flex-max, the moment of the knee maximum flexion; Before/After 0.05 s, Before/After the maximum knee flexion 0.05 s; Before/After 0.04 s, Before/After the maximum knee flexion 0.04 s; Before/After 0.03 s, Before/After the maximum knee flexion 0.03 s; Before/After 0.02 s, Before/After the maximum knee flexion 0.02 s; Before/After 0.01 s, and Before/After the maximum knee flexion 0.01 s.
















**1.5 times leg length lunge**

**the maximum lunge**

**the maximum lunge with foot external rotation**

**Figure 5.** Torque of knee in the phases of three left-forward lunges. The braking and recovery phase of the lunge was defined as the period from initial heel contact of the landing foot to toe off, starting with VGRF excessed to 10N until the right toe off the ground determined by the force plate.










**Figure 6.** Muscle activation level of knee in left-forward lunges. The time of muscle pre-activation was defined as 50 ms before touchdown, the time of muscle post-activation was defined as 50 ms after the maximum knee flexion, the braking phase was from the initial contact to the maximum knee flexion, and the recovery phase was from the maximum knee flexion time to the right toe off the ground.
